# Descemet Membrane Endothelial Keratoplasty: Intraoperative and Postoperative Imaging Spectral-Domain Optical Coherence Tomography

**DOI:** 10.1155/2015/506251

**Published:** 2015-02-26

**Authors:** Marcus Ang, Adam M. Dubis, Mark R. Wilkins

**Affiliations:** ^1^Singapore National Eye Centre, 11 Third Hospital Avenue, Singapore 168751; ^2^Singapore Eye Research Institute, Singapore; ^3^Moorfields Eye Hospital, London, UK; ^4^University College London, London, UK

## Abstract

We describe a case report of using the same handheld spectral-domain anterior segment optical coherence tomography (ASOCT) for rapid intraoperative and postoperative imaging in a case of Descemet membrane endothelial keratoplasty (DMEK). A 67-year-old woman, with Fuchs dystrophy and corneal decompensation, underwent DMEK with intraoperative ASOCT imaging using the handheld Envisu spectral domain ASOCT system (Bioptigen, Inc., Morrisville, NC, USA). We found that this easy-to-use portable system with handheld probe allowed for rapid imaging of the anterior segment during donor manipulation to visualize the orientation of the DMEK donor, as well as to confirm the initial adhesion of the DMEK donor. Moreover, the same system may be used for postoperative monitoring of graft adhesion, corneal thickness, and stromal remodeling in the clinic with very high-definition images.

## 1. Background

Corneal transplantation has evolved from full-thickness corneal grafts or penetrating keratoplasty to endothelial keratoplasty, where there is selective replacement of diseased corneal endothelium [1, 2]. Further evolution of endothelial keratoplasty techniques has led to the development of Descemet membrane endothelial keratoplasty (DMEK), where only the donor Descemet membrane (DM) with endothelium is transplanted onto the recipient cornea [3]. Studies suggest that this technique leads to faster visual recovery and a reduced risk of graft rejection, compared with Descemet stripping automated endothelial keratoplasty (DSAEK) [4, 5]. However, intraoperative challenges such as donor preparation and donor visualization within the eye have limited the popularity and uptake of the relatively newer DMEK technique [6, 7].

Anterior segment optical coherence tomography (ASOCT) allows for rapid, noncontact, high-resolution imaging of the cornea and anterior segment [8]. Recent technological developments in spectral-domain optical coherence tomography (OCT) have greatly increased imaging capabilities in terms of image resolution, compared to previous time-domain OCT technology [9]. Recently, ASOCT imaging was shown to be useful in endothelial keratoplasty, such as detection of intraoperative interface fluid [10] and postoperative follow-up after DSAEK [11]. However, the use of ASOCT to assist DMEK intraoperatively and postoperatively has not been well described.

This case report illustrates the technique of using of a handheld spectral-domain ASOCT device with rapid acquisition of anterior segment images intraoperatively for donor visualization, as well as using the same device for postoperative monitoring after DMEK.

## 2. Results

A 67-year-old woman with a history of Fuchs dystrophy underwent phacoemulsification and intraocular lens implantation (both eyes) in 2012. She presented with a gradual reduction in vision over 6 months to best-corrected vision of 6/60 in the right eye and 6/12 in the left eye. She had significant corneal edema with bullae formation in the right eye and early signs of corneal decompensation in the left eye. Intraocular pressures were normal in both eyes and her preoperative central corneal thickness was 858 ± 5.6 *μ*m and 595 ± 6.6 *μ*m in the right and left eyes, respectively.

### 2.1. Surgical Technique

Donor preparation was done using a technique previously described [6]. In summary, the donor was placed on a sterile trephination base (Coronet, Network Medical Products, North Yorkshire, UK) before the peripheral edges of the DM were scored, lifted, and peeled using nontoothed forceps, submerged in corneal storage medium. The stripped DMEK donor was then trephined to 8.5 mm and stained with trypan blue before loading into a modified glass cannula (Geuder AG, Heidelberg, Germany). Next, the recipient DM stripping was performed under air, before an inferior peripheral iridectomy was placed to prevent pupil block. The DMEK donor was injected in a double-scroll formation and gently unfolded using techniques previously described [3]. After complete unfolding, air with 20% sulfur hexafluoride (SF6) was infused into the anterior chamber below the DMEK donor at 30 mmHg for 8 minutes and released to fill two-thirds of the anterior chamber. The patient was instructed to maintain a postoperative supine position for 2 hours and reviewed to ensure that no donor dislocation or pupillary block was present. Postoperatively, the patient received one drop of dexamethasone 0.1% every 2 hours for a week and four times a day for 1 month with topical antibiotic cover. She was reviewed with ASOCT imaging at each visit: 2 hours after surgery and 1 day, 1 week, 2 weeks, and 1 month after surgery.

### 2.2. Optical Coherence Tomography Technique

**Figure 1:**
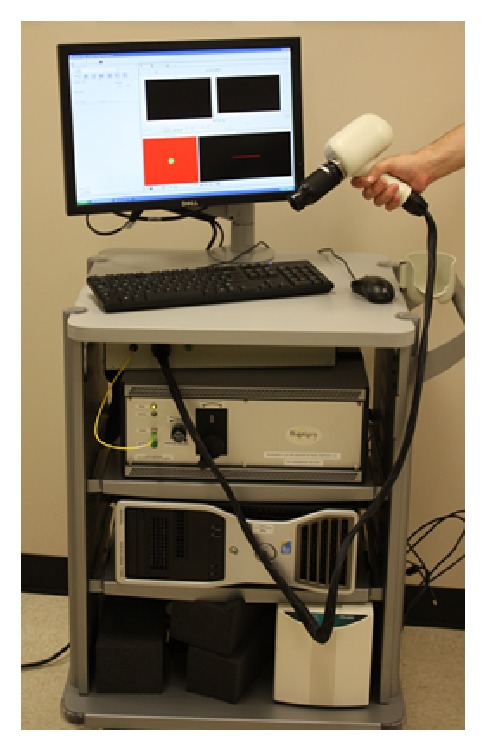
Anterior segment optical coherence tomography (ASOCT) system with handheld probe and cornea adaptation lens.

**Figure 2:**
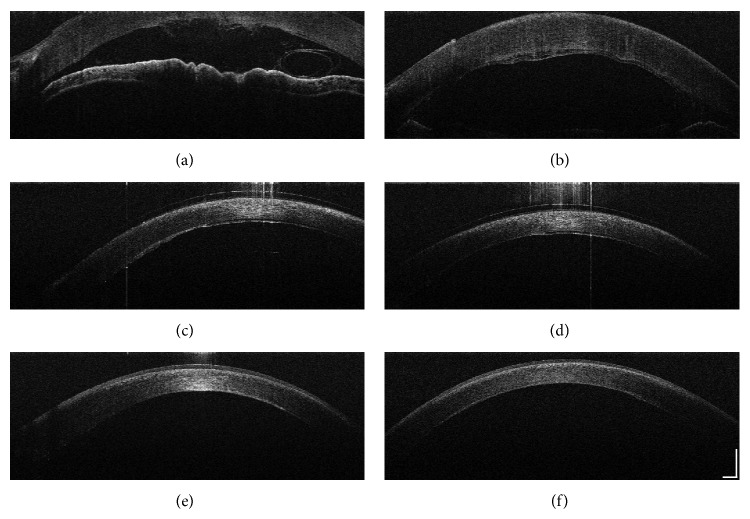
Anterior segment optical coherence tomography (ASOCT) images during and after Descemet membrane endothelial keratoplasty (DMEK). (a) Intraoperative ASOCT demonstrating single scroll configuration of DMEK donor seen in the anterior chamber. (b) Intraoperative ASOCT showing initial adhesion. (c) Two hours after DMEK showing early adhesion of DMEK donor with small amount of interface fluid. (d) One day after DMEK with further adhesion of donor graft. (e) One week after DMEK demonstrating reepithelization and thinning of central cornea. (f) One month after DMEK showing complete DMEK donor adhesion with remodeling of cornea stroma and further reduction in edema.

Intraoperative and postoperative ASOCT imaging was performed using a handheld Envisu SDOCT system (Bioptigen, Inc., Morrisville, NC, USA). The imaging system is shown in Figure 1. In short, Envisu Bioptigen SDOCT spectrometer is set for 3.2 mm deep field of view. The line-scan camera functions at 32 kHz A-line rate. The system has an adjustable with reference arm, which was optimized for anterior chamber imaging. Intraoperative imaging was done under sterile conditions. Dispersion was corrected for anterior segment imaging by an experienced imager (AMD). Scan protocols involved 10 × 10 and 12 × 12 mm rectangular volumes and high-density line-scans. Volumetric imaging consisted of 750 A-scans/B-scan and 250 B-scans/volume. These scan parameters provide for 16 *μ*m lateral by 48 *μ*m transverse resolution for the 12 × 12 mm volumetric scan and 13.3 *μ*m lateral by 48 *μ*m transverse resolution on the 10 × 10 mm volumetric scans. The operator aligned the handheld OCT probe using a sterile cover over the probe, with the perpendicular light reflex from the cornea at the horizontal and vertical center of the SDOCT aiming windows. The corneal apex was identified by a trained corneal surgeon (MA) to measure corneal thickness using calipers in company supplied InVivoVue software. Corneal thickness was defined as the distance between the anterior and posterior corneal surfaces.  Figure 2 demonstrates high-resolution intraoperative (top images) and postoperative ASOCT scans of the same patient using this portable system. From the serial ASOCT scans we were also able to estimate the corneal thickness, which noticeably reduced from the time of surgery to within 1 hour (664.3 ± 5.6 *μ*m) subsequently 1 day (572.7 ± 9.9 *μ*m) after surgery (*t*-test, *P* < 0.001). Mean corneal thickness remained stable 1 month after successful DMEK surgery at 539 ± 3.0 *μ*m, significantly improving compared to preoperative measurements (*t*-test, *P* < 0.001).

## 3. Discussion

We found that using a high-resolution handheld spectral domain ASOCT system was a useful aid to DMEK surgery, intraoperatively as well as postoperatively. The portable system with handheld probe allowed for rapid imaging of the anterior segment during donor manipulation to visualize the orientation and scroll of the DMEK donor (Figure 1, top left) as well as to confirm the initial adhesion of the DMEK donor just after 20% SF6/air injection (Figure 1, top right). To date, there has only been one other report of intraoperative OCT performed during DMEK, using a microscope-mounted OCT (iOCT; OptoMedical Technologies GmbH) [12]. However, this system performed only 10000 A-scans per second with 10 *μ*m axial resolution and image size of 3.2 mm in water, which may affect the speed of image capture, producing inverted corneal images and artifacts as demonstrated in the published images [12]. We found that the handheld system used in this report produced rapid horizontal-vertical scans that produced a video that gave the surgeon an image of the entire DMEK donor within the anterior chamber with less artifacts.

However, by using this portable ASOCT system we were able to move the system out of the operating theatre and into the clinic to monitor and scan our patient postoperatively as well. This allowed for the same, consistent high-resolution ASOCT imaging to monitor graft adhesion and reduction in corneal thickness after DMEK surgery. While older time-domain and Scheimpflug OCT systems have been shown to be not as useful for DMEK donor visualization through a hazy cornea [9], the handheld spectral domain ASOCT device used here demonstrates a very high-resolution image demonstrating graft adhesion with stromal thinning and remodeling over time. There is also visualization of corneal epithelium healing and epithelial thickness (Figure 1, bottom left and right).

The high-definition images from this handheld ASOCT system provide promising insights into the future of corneal and anterior segment imaging when dealing with fine tissues such as that in DMEK. Previous studies comparing spectral domain OCT systems (Spectralis, Heidelberg Engineering, Carlsbad, California; Cirrus, Carl Zeiss Meditec, Dublin, California; and Envisu, Bioptigen Inc., Morrisville, North Carolina) suggest that the handheld system had a low variability and high reproducibility, suggesting that the hand motion or instability of a human operator does not introduce additional error while holding the handheld probe over the target [13].

In summary, spectral domain ASOCT may be useful in providing rapid, interactive imaging that assists the surgeon to perform fine microsurgery procedures such as DMEK. The portable handheld system described here allows the surgeon to obtain consistent, high-resolution images both during the surgery and during postoperative monitoring.
